# Synthetic Germs

**Published:** 1914-06-20

**Authors:** 


### Synthetic Germs.

We have heard of synthetic sugar and synthetic rubber; it remains now to learn of synthetic bacilli. It sounds somewhat of a strange tale that was told to the *Société Internationale de la Tuberculose* at its meeting recently, but Dr. Alexandre Marey is reported to have urged that tubercle bacilli should be looked upon as new morphological products of broken-down cells. Bodies which had been obtained aseptically by the action of the tuberculin of the Pasteur Institute upon sterilised glycerophosphate of soda showed, he said, by their form and tinctorial properties, and, what one must say is much more astonishing, by their growth on potato medium and their pathogenicity to guinea-pigs, a striking likeness to the famous germ that Koch discovered. Experienced bacteriologists had taken them for the ordinary *bacillus tuberculosis*. This report, which of course would delight Dr. Charlton Bastian, that veteran upholder of bio-genesis *de novo*, will require a great deal of corroboration before it convinces. From the practical layman's point of view the experiments would seem the very type of the supererogatory and even mischievous. That is not so, of course. In science the true method of progression is that recommended to Peer Gynt—namely, to "go round." Set out with the sole object of finding a cure, and you fail. Follow up circuitous by-paths of knowledge one by one, and sooner or later success comes, the result of some thus gained indirect clue.

